# Behind the Frontline: A Review on the Impact of COVID-19 Pandemic on Healthcare Workers

**DOI:** 10.7759/cureus.29349

**Published:** 2022-09-20

**Authors:** Nikita Chhablani, Sonali G Choudhari

**Affiliations:** 1 Department of Community Medicine, Jawaharlal Nehru Medical College, Datta Meghe Institute of Medical Sciences, Wardha, IND

**Keywords:** impact of pandemic, mental health, healthcare workers, frontline workers, covid 19

## Abstract

The recent COVID-19 pandemic has been havoc that spread like wildfire, snatching the lives of many, attacking the mental health of many more, and leaving a major chunk with the constant fear of contracting this illness. It need not be mentioned that the aftermath of this pandemic was much more on frontline workers as compared to the general population. It has now become evident that the COVID-19 pandemic has influenced medical services and assets. This unpredictable mass health crisis had a significant impact on healthcare facilities, clinical transportation, patients with healthcare workers (HCWs), and their families. This difficult situation reinforces the requirement to step forward and take the necessary steps to shield frontline workers from deeds of violence and assaults. In this context, the present article reviews the impact the COVID warriors had on their physical, social, and especially psychological health. It revealed that the COVID-19 outbreak imparted a lesson greater than ever, that shielding the health and lives of HCWs is crucial to enable a better worldwide outcome. Be it doctors or nurses or paramedical staff, no one is exempt from the horrendous effect this crisis created on the mental as well as the physical and social well-being. Frontline HCWs demonstrated more degradation of these dimensions of their health and wellbeing as compared to non-frontline healthcare staff. Constant exposure to various infectious agents over the years, along with strenuous duties in the healthcare setup, led to neglected health. Being in direct contact with several COVID patients for longer durations imposed a greater risk on frontline workers. Not many studies have been done on this aspect, as health care workers are altruistically involved in saving lives rather than focusing on their own health. Efforts should be made to have a closer look at the aftermath of this havoc on HCWs and go into the depth of depredation in order to better manage any forthcoming healthcare crisis without hampering the well-being of HCWs.

## Introduction and background

The COVID-19 pandemic has caused significant strain on the already overburdened health care system. It has pushed physicians to the brink of their clinical abilities while also taking a significant toll on their physical and psychological well-being [1]. The incidence of COVID-19-related health effects on healthcare workers (HCWs) has not been documented due to methodological limitations of studies; none of the studies could adequately demonstrate the impact of this pandemic on HCWs, particularly because it is difficult to locate the actual denominator of data. Furthermore, studies that are interventional in nature are very few. More research on the raging issue can help in the estimation of the risk involved and the means to deal with it [2].

HCWs were on the frontline of this crisis, actively engaged in providing care and comfort for COVID-19 patients while coping with crucial issues on an everyday basis like lack of hospital infrastructure, scarcity of oxygen supply, ventilator support, personal protection equipment, laborious working hours, the danger of contracting the disease and spreading the virus [3]. This emerging pandemic is taking a toll on the well-being of frontline workers in all domains of health. It has now become a high priority to comprehend controllable factors responsible for the poor mental well-being of HCWs and alter them in their favor [4]. Many mental health issues were being faced by the frontline workers such as long working hours, loneliness, inadequate rest and self-care, variability of disease pattern, feeling of helplessness, dealing with the pain of losing multiple patients every day, facing violence, depression, post-traumatic stress disorder, the decline in private practices, the decline in elective procedures, increased burden on resident doctors, uncertainty regarding exams, loss of academics, prolongation of course duration, lack of clinical exposure of other medical ailments, lack of learning experience in their respective fields, inability to visit family members, fear of spreading disease to family members, and facing the unknown [5,6]. The frequency and intensity of experiencing such signs and symptoms by frontline COVID warriors were greater than quadruple in comparison to pre-COVID 19 times. Disruption in the health care system and medical education has also been observed globally [7]. Physicians around the world had to modify their ways to treat patients, prioritizing patients and facing challenges due to limited resources, even withdrawing and withholding potential lifesaving treatment. HCWs had to weigh the risks associated with aiding COVID patients, along with balancing the workplace and responsibilities of high-risk family members. The obligation of isolating themselves if they have COVID-19-like signs and/or symptoms takes them away from being on the frontline. All such factors have contributed to a feeling of guilt, stress, and moral dilemmas. When clinicians had to make such decisions that contradict their moral and professional commitments, they face the challenging situation of knowing the requirement of the patient but being unable to provide that care due to restrictions beyond their control. Several changeable elements have been related to the maximum extreme degree of psychiatric signs and symptoms: the scarcity of adequate personal protective equipment, the inadequacy of preparation for the workplace, communication, training, and the excessive load of work [8]. HCWs experience work-related challenges and face an ethical dilemma, in addition to well-known risks they deal with daily as general physicians [9].

Working on the frontline, being an allied HCW or supervisor, preceding psychiatric diagnoses, and stressful and traumatic events have been additionally substantially related to extreme psychiatric signs and symptoms. Sharing fears, resilience, and moral guidance for remedial strategies have been related to lesser psychiatric symptoms. As coronavirus disease is a newly re-surfaced contagious disease, fear, panic, stress, and dilemma spread quickly even before the containment of the spread of the disease [10].

This pandemic has aggravated the tension between the medical profession and clinicians' well-being. Mental health is not the only aspect that is affected due to this pandemic but it has also struck physical health. Flu-like symptoms create a sense of fear, and those who have pre-existing comorbidities face more issues while performing COVID duties. The use of personal protective equipment for a longer duration leads to uneasiness, excessive sweating, discomfort, suffocation, dehydration, nutritional deficits, and fungal infections [11].

The present review is undertaken to determine the influence of the COVID 19 pandemic on HCWs, especially their psychosocial health, to study the components associated with the impact of the pandemic on healthcare delivery, and to identify the efforts of HCWs in minimizing the blow of COVID 19.

## Review

Methodology

**Figure 1:**
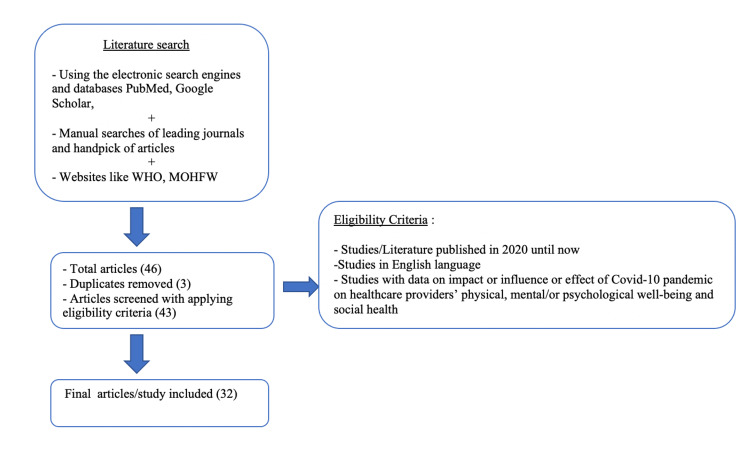
Study selection

Relevant articles were selected after a thorough search using PubMed, Google Scholar, and manual searches of leading journals including websites like WHO, and the Ministry of Health and Family Welfare (MoHFW). The following keywords were used: healthcare workers, COVID-19 pandemic, stress, mental health, COVID warriors, impact, effect, and frontline workers. Studies published in the English language from 2020 until now were included. A total of 46 articles were found, out of which around 32 were included that fulfilled the eligibility criteria completely (Figure 1).

Discussion

It has been reported that junior medical trainees are profoundly affected by this raging pandemic and despite putting their own lives at stake and their best efforts to save a life, HCWs are facing vandalism and abuse [12]. The vandalism towards physicians and other clinical personnel has expanded in the course of the last couple of decades, with up to 75% of the specialists confronting this while practicing in India. Clinicians attribute the rise in violence towards HCWs to a blend of oblivion and dread, which is now intensified by the epidemic. The lockdown has aggravated the issue, with patients and their relatives not being able to get timely medical services because of suspension of transport facilities, irritability following isolation, or limitations of containment areas. The kinds of assaults have now gone from threats, abuse, and intimidation to obnoxious attacks. However, there have additionally been reports of abduction, misconduct, and murder [13]. The explanations behind viciousness against medical personnel might fluctuate from uneasiness, alarm, dread, and lack of information (with regards to the spread of SARS-CoV-2 infection among the population), doubt, and misplaced statements on social media platforms [13].

Front-liners are constantly dealing with external as well as internal and emotional turmoil. None of the education and experience in healthcare prepared frontline workers to deal with the grief of losing so many patients at once, feeling of helplessness, and getting blamed for the inevitable and helpless situation.

HCWs are critical to an efficient response to the COVID-19 outbreak. They have vital interests in diagnoses, treatment, and containment, and their dedication to dealing with enhanced risks is essential for a better global health outcome. HCWs had been bearing excessive workload, risks of infection, and pressure from next of kin to satisfy their healthcare demands. Despite everything, these conventional public health morals have done a bare minimum for the safety of the rights of HCWs [14].

Since the beginning of the pandemic, HCWs have been shown great support, harmony, and gratitude that they ever have. Yet, assaults on HCWs have constantly been mentioned and encompass incidents associated with the COVID-19 epidemic worldwide. HCWs working on the frontline have not only had to cope with a venture of seeking to fulfill their obligations contingent on the inclusion of the public (interdependence of attempt in reaching objectives) but additionally, also living up to those great expectations and stereotypes of being a superhero (e.g., strong, unfazed) [15].

**Figure 2:**
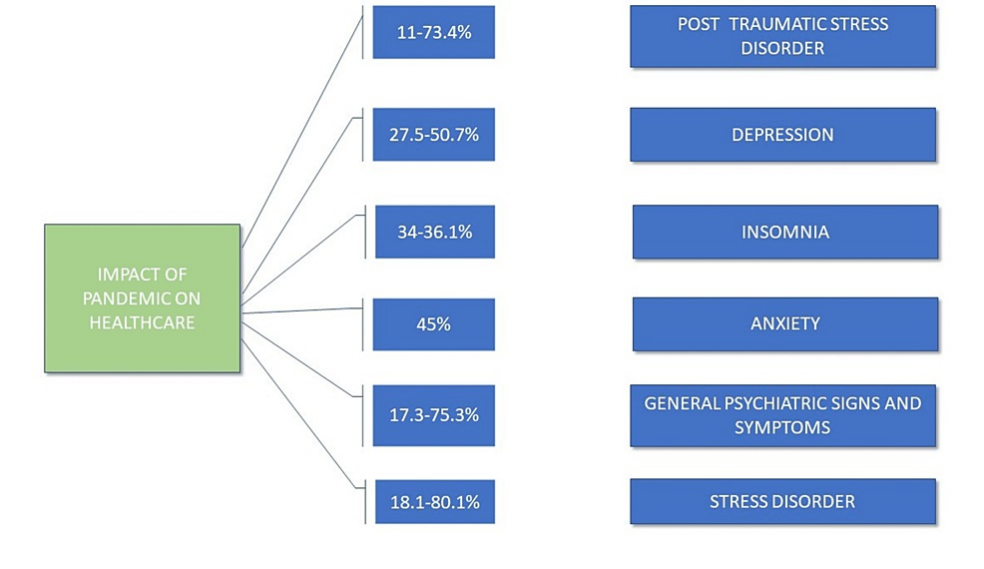
Impact of COVID-19 on healthcare workers (HCWs) [19]

The sharp rise of cases and the disease’s expanding geographical spread raises severe concerns about the future trajectory of the ever-growing pandemic [16]. HCWs, at the core of the daunting emergency of COVID-19, confront demanding situations while providing care and treatment to patients of COVID-19 such as decreasing the transmission of disease, creating reasonable short-term methodologies, and making long-term strategies. Clinicians, coordinators, and investigators have an improved sense of purpose and determination to use science and technology to resolve problems that are crucial to patients globally [17]. HCWs must proceed to effectively treat non-COVID patients and keep up individual obligations while protecting themselves and their families. The mental burden and well-being of HCWs have obtained increased awareness, with studies persevering on excessive rates of burnout, work-related stress, and suicide [18]. According to a study [19], between 11% and 73.4% of HCWs, mainly comprising internal medicine doctors, emergency medicine doctors, nurses, and paramedical staff, reported post-traumatic stress disorder symptoms, lasting from one to three years in 10% to 40%. Depression-like episodes and related symptoms are reported in 27.5% to 50.7%, insomnia symptoms in 34% to 36.1%, and severe anxiety symptoms in about 45%. General psychiatric signs and symptoms during a pandemic range from 17.3% - 75.3%. A high range of work-related stress is reported in 18.1% - 80.1% (Figure 2).

Empirical evidence emphasizes the importance of addressing the adverse effects of pandemics on the mental and physical health of HCWs. Assessing and advertising coping techniques, utmost concern for HCWs working on the frontline, availability of adequate protective essentials, and organization of online supportive services should be among the recommendations [19]. Social distance policies, compulsory lockdowns, quarantine periods, and the fear of contracting the disease, along with the suspension of efficient activity, fear of the future, and loss of income, all affected citizens’ and workers’ physical as well as mental health. Factors related to the workplace can significantly determine whether people’s mental health improves or worsens as a result of the epidemic [20].

There is a high possibility that HCWs would be having an excessive amount of stress over the chance of getting infected with COVID-19, regardless of sporting heavy personal protective gear. The lack of healthcare attendants being concerned with warmth, maintaining inter-personal social distance measures, and sporting unique protective equipment long-term has resulted in a shift in universal clinical behaviors [21]. It is a possibility that even after the containment of COVID-19, pandemic prevention protocols might nonetheless be carried out. This would promote pandemic-preventable medical behaviors such as frequent hand washing, use of masks while visiting hospitals, emphasizing the history of contact, and social distance practices. Due to the complex process of preventing pandemics, the waiting time to see a physician might increase, resulting in longer stay time in the hospital. All of this will affect the quality of the medical system. Indeed, this pandemic has brought massive diversity in doctor-patient relationships [22].

This is no surprise that much exploration has already been done to comprehend the effects of being on the frontline. The distressful impacts of this type of work are noted to be associated with such control, which is externally situated. Components of the work in our profession demand interdependency to make optimal clinical decisions, and, as a result of which, the hold-over consequences are not independent. In this COVID-19 pandemic, interdependency is crucial. Frontline warriors are relying on the general population to stick to public health guidelines to forestall coronavirus transmission, reduce the distressing condition of their work, and reduce their potential workload. Notably, at some point in this situation, we have witnessed visible proof for the broader socio-cultural surroundings having a crucial role in HCWs emotions of praise for their efforts [23]. Keeping the rewards aside, which front-liners undoubtedly deserve for their tireless, unwavering efforts, most health care facilities struggled to provide them with adequate personal protective equipment, adequate supply of quality masks, and efficient health services in case of contracting COVID infection [24]. Lack of well-preparedness is a major contributing factor to the struggles faced by HCWs globally [25]. Amidst such adverse conditions, while HCWs work tirelessly to protect individuals, families, and communities with limited supplies, they have become unanticipated targets in this battle against COVID-19. Several incidents of violence against them have been reported in India during such unparalleled times. Determination of an exact number of such incidents is not possible, yet the value of the lives of doctors in India is a matter that must be pondered upon. 

Table 1 lists the key findings of studies investigating the impact of COVID-19 on HCWs.

**Table 1:** Key findings of studies investigating the impact of COVID-19 pandemic on healthcare workers (HCWs)

Author	Year	Outcome/Results
Misra-Hebert et al. [2]	2020	The patient-facing HCW had higher odds of SARS-CoV-2 infection. Healthcare system with significant risk-mitigation strategies to prevent the spread of COVID-19 infection and other similar infectious agents play a crucial role.
Kalaitzaki et al. [3]	2020	Evidence-based interventions aiming at safeguarding health professionals from the negative effects of the pandemic, while concurrently -and mainly- strengthening their personal assets and resources, are of paramount importance both for the safety of professionals and patients, and the quality of patient care itself.
Gilleen et al. [4]	2021	Poor mental well-being was prevalent during the COVID-19 response, however, controllable factors associated with severe psychiatric symptoms are available to be targeted to reduce the detrimental impact of COVID-19 and other pandemics on HCW mental health.
Lakhani et al. [8]	2020	HCWs are working day and night just to protect the citizens despite being at high-risk exposure and they are being aimed by the virus due to shortage of Personal Protection Equipment kits. They are also being brutally harassed by the patients themselves. Social, economic, psychiatric and many other factors are responsible for deteriorating the health of these frontline HCWs who are now being allegedly regarded as "Healthcare Warriors".
Shu-Ching et al. [10]	2020	COVID-19 highlight the risk of safety problems for healthcare providers and nurses. Manpower shortages during infectious disease outbreaks may be caused by uncertainties regarding life-threatening infectious sources and real cases of infection among healthcare staff.
Gautam et al. [24]	2020	There is an urgent need for a system-level approach to address the issues that COVID-19 has created to better protect and safeguard our medical workforce for the future. The medical profession, health systems, and society all have a part to play in ensuring this support is provided. Individual doctors need to be empowered to recognise their own limitations as well as their wellbeing and support needs.
Shaukat et al. [26]	2020	The frontline HCWs are at risk of physical and mental consequences directly as the result of providing care to patients with COVID-19. Even though there are few intervention studies, early data suggest implementation strategies to reduce the chances of infections, shorter shift lengths, and mechanisms for mental health support could reduce the morbidity and mortality amongst HCWs.
World Health Organization, Geneva [27]	2021	A population-based estimate indicates that around 115 500 HCWs out of the global health and care workforce of 135 million people could have lost their lives. This is an alarming picture of the impact of the pandemic on HCWs who need to be provided with better protection (including access to vaccines, personal protective equipment, training, testing and psychosocial support) and decent work conditions (including adequate remuneration and protection against excessive workloads).
Hall [28]	2020	The rapid spread of the disease created challenges for healthcare systems and forced HCWs to grapple with clinical and nonclinical stressors, including shortages of personal protective equipment, mortality and morbidity associated with COVID-19, fear of bringing the virus home to family members, and the reality of losing colleagues to the disease.
Gupta et al. [29]	2201	During COVID-19 crisis, doctors face several challenges in treating patients with COVID-19. The psychological burden and overall wellness of HCWs have received heightened awareness, with research continuing to show high rates of burnout, psychological stress, and suicide. Anxiety and stress were significantly increased, leading to negative impacts on both self-efficacy and sleep.

A study [30] depicting the transmissibility of COVID-19 has revealed that a contaminated patient will affect two other persons during the beginning stage of pestilence. It is incredibly challenging to anticipate a specific population likely to be influenced sooner rather than later. Considering a global trend, we can undoubtedly say that the medical care needed to be made to the COVID epidemic will surpass our ability. India comprises 11,54,686 enlisted specialists in the strength of present-day medicine. Presently, a single government general physician considers the need of 10,926 people. As of now, 60% of the population of India lives in rural India. To serve sufficient medical care to the population of rural India, the public authority has set up 25,743 primary health care centers, 1,58,417 sub-centers, and 5,624 community health centers. Currently, 7,13,986 beds are accessible in government emergency clinics in India which adds up to 0.55 beds per thousand population. A few states like Bihar, Gujarat, Odisha, Jharkhand, Madhya Pradesh, Assam, Haryana, Maharashtra, and Manipur, which comprise over 70% of the total population of India, have a population: bed ratio even below public normal, yet a few states like Tamil Nadu, Kerala, and Sikkim has the better population to bed proportion.

Implementation of the subsequent techniques may also reduce the weight of health outcomes: the appropriate education and training on the usage of protective gears, strict contamination management protocols, shorter duration of shifts, and provision of psychological health and aids [26]. Triumphing over this pandemic seems to be a far-off prospect; working to reduce casualties and attenuate risks is the principal means now [31].

WHO has pointed out that an effective surveillance system, dedicated instruments, standardized measurements, and investigations are needed to ensure monitoring of the impact of COVID-19 on HCWs [27]. In addition, healthcare stakeholders should create short- and long-term plans to support the mental health of workers during and after the COVID-19 pandemic [28]. As Bergman et al. state, we do not need social distancing, but physical distancing with social connectedness [32]. It is the need of the hour to generate more awareness among HCWs and specific screening strategies to be implemented for the frontline workers. Further long-term studies focusing on their mental health should be planned, as adverse mental health conditions will further affect them as the pandemic advances [29].

## Conclusions

Nobody could have predicted the enormous blow on our planet by a microscopic virus in the span of months. The novel coronavirus has a wide-ranging effect on the health of people worldwide. Being in the frontline, HCWs are taking the first blow, constantly getting hit by the diversified nature of this illness and challenging the adversities coming their way. The mass casualties caused due to COVID-19 are evident in front of our eyes; the hidden fact is how our overburdened, unrested COVID warriors are turning into COVID worriers while working day and night tirelessly to reduce these casualties. Doctors, nurses, and paramedical staff members of all the disciplines comprising every stratum in the health care system hierarchy have been at the frontline for 18 months. Understanding the pandemic's impact on those responsible for decreasing its impact globally is crucial. Improvement in healthcare facilities, procurement of medical aid, equipment, and protective gear, and advancement of ambulance services is need of the hour to better manage healthcare crises in the future. Observational fact underlines the need to deal with the negative impacts of pandemic flare-ups on HCWs' psychological and physical well-being. Suggestions should incorporate the appraisal and advancement of adapting methodologies, special consideration to the frontline HCWs, arrangement of satisfactory defensive essentials, and arrangement of online supportive administrations. It is the need of the hour to save the saviors, to manage the situation better, to understand if it is the incapability of the front liners or inadequacy of the system and scarcity of resources, and to understand what is happening behind the closed doors of ICUs. Mass casualties are now following a decreasing trend, and we have to decide if the efforts of these saviors behind this should be appreciated with praise and respect or with violence and assault.
